# Integrating biomarkers across omic platforms: an approach to improve stratification of patients with indolent and aggressive prostate cancer

**DOI:** 10.1002/1878-0261.12348

**Published:** 2018-08-07

**Authors:** Keefe Murphy, Brendan T. Murphy, Susie Boyce, Louise Flynn, Sarah Gilgunn, Colm J. O'Rourke, Cathy Rooney, Henning Stöckmann, Anna L. Walsh, Stephen Finn, Richard J. O'Kennedy, John O'Leary, Stephen R. Pennington, Antoinette S. Perry, Pauline M. Rudd, Radka Saldova, Orla Sheils, Denis C. Shields, R. William Watson

**Affiliations:** ^1^ UCD School of Mathematics and Statistics University College Dublin Ireland; ^2^ UCD School of Medicine Conway Institute of Biomolecular and Biomedical Research University College Dublin Ireland; ^3^ Department of Histopathology Central Pathology Laboratory Trinity College St James Hospital University of Dublin Ireland; ^4^ School of Biotechnology Dublin City University Ireland; ^5^ Biomedical Diagnostics Institute National Centre for Sensor Research Dublin City University Ireland; ^6^ Prostate Molecular Oncology Institute of Molecular Medicine Trinity College Dublin St James Hospital Dublin Ireland; ^7^ NIBRT GlycoScience Group National Institute for Bioprocessing Research and Training Dublin Ireland; ^8^ Cancer Biology and Therapeutics Lab UCD School of Biomolecular and Biomedical Science University College Dublin Ireland

**Keywords:** biomarkers, indolent, integration, LASSO, omics, prostate cancer

## Abstract

Classifying indolent prostate cancer represents a significant clinical challenge. We investigated whether integrating data from different omic platforms could identify a biomarker panel with improved performance compared to individual platforms alone. DNA methylation, transcripts, protein and glycosylation biomarkers were assessed in a single cohort of patients treated by radical prostatectomy. Novel multiblock statistical data integration approaches were used to deal with missing data and modelled via stepwise multinomial logistic regression, or LASSO. After applying leave‐one‐out cross‐validation to each model, the probabilistic predictions of disease type for each individual panel were aggregated to improve prediction accuracy using all available information for a given patient. Through assessment of three performance parameters of area under the curve (AUC) values, calibration and decision curve analysis, the study identified an integrated biomarker panel which predicts disease type with a high level of accuracy, with Multi AUC value of 0.91 (0.89, 0.94) and Ordinal C‐Index (ORC) value of 0.94 (0.91, 0.96), which was significantly improved compared to the values for the clinical panel alone of 0.67 (0.62, 0.72) Multi AUC and 0.72 (0.67, 0.78) ORC. Biomarker integration across different omic platforms significantly improves prediction accuracy. We provide a novel multiplatform approach for the analysis, determination and performance assessment of novel panels which can be applied to other diseases. With further refinement and validation, this panel could form a tool to help inform appropriate treatment strategies impacting on patient outcome in early stage prostate cancer.

**Abbreviations**
AUC: area under the curve
DRE: digital rectal examination
IGP: Integrated Geometric Pooling
ORC: Ordinal C‐index
PCa: prostate cancer
PCRC: Prostate Cancer Research Consortium
ROC: receiver operating characteristic

## 1 Introduction

Prostate cancer (PCa) is the leading noncutaneous diagnosed cancer in men, but the majority of men die with PCa rather than from it indicating that most tumours are indolent not progressing during the patient's life span. These tumours based on the National Comprehensive Cancer Network classification system (Mohler *et al*., 2010) are characterised by their clinical behaviour as indolent, slow‐growing tumours. However, PCa can also be aggressive and fast‐growing with lethal progression (Penney *et al*., 2011). The ability to distinguish between indolent and aggressive disease would improve on the selection of appropriate treatments and impact on patients’ outcomes and quality of life.

Tumour heterogeneity and multifocality of PCa represents one reason for the difficulty in distinguishing these disease types (Oon *et al*., 2011). Single biomaterial‐based tests such as Prolaris and Oncotype DX prostate (Murphy *et al*., 2015) have shown some improvements over existing models (Cooperberg *et al*., 2013; Klein *et al*., 2014) but are dependent on sampling the correct site. It is clear that additional features, types of biomaterial to be sampled and methods of tumour classification are needed to guide disease management at the time of diagnosis and to prevent over‐treatment of indolent disease. We hypothesised that multivariate models built from different omic data (DNA methylation, transcriptomic, proteomic and glycomic) across different biomaterial (tissue, serum) may lead to superior accuracy for PCa risk stratification over individual disease features. Recent studies have combined genomic instability and the micro‐environment factor hypoxia, with clinical parameters to improve prognostication in patients with localised disease to predict biochemical recurrence (Lalonde *et al*., 2014).

Building on previous work of the Prostate Cancer Research Consortium (PCRC), our study undertook to prove the concept that integrating data from different omic platforms assessed in different biomaterial (serum and tissue) in a single cohort of patients with prostate cancer could identify a panel of biomarkers that could distinguish between indolent and aggressive PCa with improved performance compared to individual platforms alone. We evaluated preselected panels of biomarkers across a number of omic platforms from a cohort of 158 patients with pathologically indolent, significant and aggressive PCa. These panels included DNA methylation, coding and noncoding transcripts, protein and glycosylation biomarkers, and our study undertook to identify an integrated panel with the ability to distinguish between indolent localised disease and aggressive nonlocalised disease. We developed a statistical approach for the integration of multiple omic data sets, handling missing data in a multiclass context and assessment of three performance parameters of area under the curve (AUC) values, calibration and decision curve analysis (Klein *et al*., 2014) which can be applied to other cancers and diseases. Through this, the study yielded a panel of DNA methylation, coding and noncoding transcripts, protein and glycosylation biomarkers which predict indolent localised disease with a high level of accuracy which with further refinement could form a tool to help to inform appropriate treatments impacting on early patient outcome and quality of life.

## 2 Materials and methods

### 2.1 Clinical cohort and samples

#### 2.1.1 Clinical cohort and patient characteristics

A cohort of 158 radical prostatectomy PCa patients were recruited following written consent as part of the Irish PCRC bioresource which were collected from 2004 to 2010 conforming to the standards set by the Declaration of Helsinki from three tertiary referral hospitals for newly diagnosed PCa with no prior treatment and identified as suitable for surgical treatment. Blood and tissue samples were collected in accordance with standard operating procedures. Patients were assigned based on their final histopathology applying the Epstein criteria, to: indolent or low‐risk disease defined as organ confined Gleason score 6 with no grade 4 (*N* = 46); significant or intermediate risk defined as the presence of Gleason grade 4 but organ confined (*N* = 56); and aggressive disease (*N* = 56) defined as the presence of Gleason grade 4 and extra prostatic disease. Patients were matched for age and pre‐operative PSA (Table S1 for patients’ clinical details). Power analysis was conducted in accordance with Ferriera and Zwinderman (2006).

Ethical approval was granted from the respective ethics committees at the Mater Misericordiae University Hospital, St James Hospital and Beaumont Hospital.

#### 2.1.2 Tissue sample preparation

Tissue sections were reviewed by a pathologist who identified areas of tumour for macrodissection which was placed in an ‘RNase/DNase‐free’ Eppendorf tube. DNA and total RNA were isolated using RecoverAll Total Nucleic Acids Isolation kit (Ambion, ThermoFisher Scientific, Waltham, MA, USA) and quantified by Qubit fluorescence (Promega, Madison, WI, USA).

#### 2.1.3 Serum samples

Pre‐operative blood samples were collected under standard operating procedures and underwent no more than two freeze/thaw cycles prior to analysis.

### 2.2 DNA methylation tissue analysis

DNA was subjected to sodium bisulphite conversion using the EpiTectFast Bisulfite Modification kit (Qiagen, Manchester, UK). Briefly, DNA methylation at 17 differentially methylated regions identified from previous unpublished studies in independent cohorts was studied by quantitative methylation‐specific PCR, as previously described (Perry *et al*., 2013). For each assay, oligonucleotides were designed to span multiple CpG dinucleotides.

### 2.3 mRNA and miRNA tissue analysis

#### 2.3.1 cDNA synthesis for mRNA expression analysis

cDNA was synthesised using the High Capacity cDNA Reverse Transcription Kit (Applied Biosystems, Dublin, Ireland).

#### 2.3.2 cDNA synthesis for miRNA expression analysis

cDNA for use in miRNA expression analysis was synthesised using the TaqMan© miRNA Reverse Transcription Kit (Applied Biosystems). miRNA was assessed by two platforms depending on the panel as detailed below and are included in the two separate Transcript 1 and Transcript 2 data blocks due to the scaling of the data.

#### 2.3.3 PCR ExiLENT SYBR^®^ analysis

For the Transcript 1, panel miRNA profiling was performed by Exiqon Services (Denmark). RNA was reverse transcribed using the miRCURY LNA™ Universal RT miRNA PCR, Polyadenylation and cDNA synthesis kit (Exiqon). cDNA was assayed using ExiLENT SYBR^®^ Green master mix and LNA primers. PCR amplification was performed in a LightCycler^®^ 480 Real‐Time PCR System (Roche, Dublin, Ireland).

#### 2.3.4 Custom TaqMan^®^ Low Density Arrays (TLDAs) with pre‐amplification

TaqMan^®^ gene and miRNA low‐density 384‐well arrays were performed according to manufacturer's guidelines for the assessment of Transcript 2 panel. Briefly, arrays were designed with custom hybridised probes using the Applied Biosystems protocol.

### 2.4 Proteomics Multiple Reaction Monitoring (MRM) design and data acquisition of serum analysis

All MRM assay design and data analysis was conducted using skyline software (V.2.5.0.6157; University of Washington, Seattle, WA, USA) using previously reported criteria for proteotypic peptide selection. MRM data were acquired using an Agilent 6460 mass spectrometer coupled to a ChipCube interface with a C18 nano‐LC Chip (Agilent G4240‐62010) (Agilent Technologies, Dublin, Ireland). MRM data were analysed in Skyline using an external peptide library database (http://www.peptideatlas.org) and with in‐house spectral libraries generated from previous serum LS‐MS/MS discovery experiments and from synthetic peptides. Dried peptide samples were reconstituted, and prior to analysis, serum samples were subjected to trypsin digestion using a method adapted from the FASP protocol (Wiśniewski *et al*., 2009). The pre‐analytical reproducibility of serum digestions was measured and shown to be highly reproducible by MRM quantification of proteotypic peptides representing the 14 most abundant serum proteins which between them comprise > 95% total serum protein. Patient samples were analysed using liquid chromatography–mass spectrometry/mass spectrometry in randomised order in batches. To establish the reproducibility of multiple reaction monitoring (MRM) analysis across each batch and between batches, a technical replicate sample was used. Furthermore, evaluation of possible matrix effects was analysed by experiments in which synthetic peptides were spiked into serum samples (Rogatsky and Stein, 2005). The chromatographic resolution and signal/noise ratios for individual peptides revealed that matrix effects did not interfere with peptide measurements. Analysis reports for each batch of samples were exported from Skyline for further evaluation of discriminating candidate biomarkers.

### 2.5 N‐Glycan serum analysis

Glycoprotein denaturation and N‐glycan release was carried out in a Hamilton Robotics Star Let liquid‐handling platform as previously described (Royle *et al*., 2008). 2‐AB labelled N‐glycans were separated by ultra‐performance liquid chromatography with fluorescence detection on a Waters Acquity ultra‐performance liquid chromatography H‐Class instrument with a fluorescence detector and empower software (Waters, Dublin, Ireland). The system was calibrated using an external standard of hydrolysed and 2AB‐labelled glucose oligomers to create a dextran ladder, as described previously by Royle *et al*. (2006).

### 2.6 Modelling and statistical analysis

#### 2.6.1 Modelling

A multiblock approach was taken to integrate the various data blocks into a single model by aggregating the probabilistic outputs of each separately modelled block. Under this analysis, the DNA methylation, Transcript 1, Transcript 2, proteomics and glycosylation datasets are considered as separate blocks. Clinical data are treated as a constituent block like any other. It includes Age; PSA (recorded continuously without thresholding); Gleason Score at biopsy (as an ordinal factor with levels 6, 3 + 4, 4 + 3 and ≥ 8); and digital rectal examination (DRE).

Rather than imputing the missing DRE values, DRE was coded as missing (27%), abnormal (23%) or normal (50%), with missing as the baseline reference category, as proposed by Formann (2007), because we considered that missingness of DRE may be informative. Furthermore, we avoid the sensitivity of imputation methods to assumptions about the missingness mechanism. Otherwise, all blocks contained full information.

Preprocessing is applied to the omic blocks: the square‐root transform on the methylation data, the log transform on the Transcript 1 and Transcript 2 blocks, the centred log ratio transformation on the glycosylation data, to standardise the composite peak heights (Aitchison, 1982), and finally, for the proteomic data, peptides with large amounts of undetected values are recoded as simply present or absent.

Apart from the protein biomarkers, multinomial logistic regression is used to model each individual block against the indolent, significant and aggressive disease types. This models the log‐odds ratio of significant disease and log‐odds ratio of aggressive disease, relative to the log‐odds ratio of indolent disease as a linear function of the biomarkers (Hastie *et al*., 2009). The model equation (Equation S1) is used to estimate the probability of indolent, significant and aggressive disease for each patient, which are ultimately treated as inputs to the final aggregate model.

For the proteomic block, quasi‐perfect separation occurred using logistic regression (Albert and Anderson, 1984); to deal with this and overcome issues of overfitting, LASSO regularisation is used on this data only (Hastie *et al*., 2009; Heinze and Scheraper, 2002). This also achieves variable selection: the proteomic panel is treated as those variables with non‐negative LASSO coefficients. For the logistic regression models fitted to all other blocks, forward and backward stepwise variable selection algorithms using Akaike Information Criterion are employed to compare models with different marker combinations (Hastie *et al*., 2009); this penalises model complexity, in order to refine each block to the most parsimonious biomarker panels (Table 1).

**Table 1:** Variables retained in biomarker panel

Data set	No. variables	No. retained	Biomarker panels
Clinical data	4	4	bxGS, Age, PSA, DRE
DNA methylation	17	4	GSTP1, CTNNA2., MAGPIE.1, LXN
Transcripts 1	12	4	miR.663a., miR.20a.5p, miR.221.3p, miR.143.3p
Transcripts 2	25	9	miR.330, miR.222, miR.101, miR.16.1, ALCAM, FAM49B, IGFBP3, AMACR, SFRP4
Proteomics	91	27	DYVSQFEGSALGK and LLDNWDSVTSTFSK (APA1), EPCVESLVSQYFQTVTDYGK (APOA2), IDQNVEELK(APOA4), NPNLPPETVDSLK (APOD), WVQTLSEQVQEELLSSQVTQELR and VQAAVGTSAAPVPSDNH (APOE), DLLLPWPDLR and VAAGAFQGLR (LRG1), ITCAEEGWSPTPK and TGDIVEFVCK (CFHR2), SDLAVPSELALLK and AAIPSALDTNSSK (LGALS3BP), SVLGQLGITK (SERPINA1), ADLSGITGAR (SERPINA3), NEDSLVfVQTDK (A2M), DFDFVPPVVR (C3), TEHYEEQIEAFK (C9), ELGCGCAASGTPSGILYEPPAEK (CD5L), EDSLEAGLPLQVR (CHGA), TTLSGAPCQPWASEATYR (F12), YGIDWASGR (FCN3), LAAIAESGVER (PSMB6), ETLLQDFR (AMBP), WEAERPVYVQRP (AZGP1), NVPLPVIAELPPK (IGHM), EAVPEPVLLSR (TGFB1)
Glycosylation	50	13	A2[3]G1S[3]1, A2G2S[3]1, A1, A2BG2S[3,6]2, A4F1G3S3, M6 D3, A2BG2S[6]1, A2[6]BG1, A4G4S[3,3,3,3]4, FA2BG2, A3G3S[3,3,6]3, FA2[6]BG1, FA2G2S[3,6]2

Table includes within each dataset the number of variables measured, the number retained by the stepwise selection procedure, and the final biomarker panels used by the individual logistic regression models. For the Proteomics variables, the peptide sequence is included with the gene name in brackets. See Table S3 for further details on function and relevance to PCa.

Cross‐validation was used to tune the LASSO penalty for the proteomic panel. In addition, leave‐one‐out cross‐validation is used on each model after variable selection to obtain accurate, unbiased predicted probabilities for each patient.

#### 2.6.2 Geometric pooling

The predicted probabilities are aggregated according to the geometric pooling formula (*1) below (also see Equation S2) where *i* denotes patient identity, *t* denotes disease type and *b* denotes the biomarker block, such that *B*_*i*_ represents the available blocks of information for the *i*th patient, *X*_*i,b*_ represents data for the *i*th patient from block *b*, and *M*_b_ represents the model for the *b*th block of data. This constitutes an ensemble system whereby individual biomarker panels and their associated models vote according to a weighted combination of their predicted probabilities. Therefore, if biomarkers of different types measure different biological activities and perform well in doing so, one model could help correctly classify a patient that has been misclassified by another, by having a stronger contribution in the aggregation process if it is more confident in its classification. Conversely, misclassifications from individual models will have a weaker contribution if they have low probability.

$$\begin{array}{cc} P\left({\text{Type}}_{i,t}|X_{i,\mathrm{Agg}},\;M_{\mathrm{Agg}}\right) & \propto \prod_{b\;\in \;B_{i}}\left(\frac{P({\text{Type}}_{i,t}\left|X_{i,b},M_{b}\right)+\varepsilon }{P({\text{Type}}_{i,t}\left|M_{b}\right)}\right)\\ & \times \prod_{b\;\in \;B_{i}}P{\left({\text{Type}}_{i,t}|M_{b}\right)}^{\frac{1}{|B_{i}|}}\\ & \propto \prod_{b\;\in \;B_{i}}\left(\frac{P({\text{Type}}_{i,t}\left|X_{i,b},M_{b}\right)+\varepsilon }{P({\text{Type}}_{i,t}{\left|M_{b}\right)}^{1-\frac{1}{|B_{i}|}}}\right) \end{array}$$

The denominator here represents the unequal prior prevalences of indolent, significant or aggressive disease in each block (Table 2), whereas the numerator represents the posterior predicted probability of that disease type according to each individual model. The method therefore computes the probability of a particular disease type for a given patient, by multiplying the predicted probabilities for that disease type, for each model for which there exists information for that patient, dividing by the unequal prior prevalences of that disease type in each block, and then multiplying this quantity by the geometric mean of the prevalences in blocks which are available for that patient. Finally, the outputted probabilities are normalised to uncover the proportionality constant. A small zero‐correction term, ε, is added to the numerator inside the product to ensure that predicted probabilities of exactly zero arising from any individual models do not subsequently dominate the product. This is set to the inverse of the product of the sample size (*n* = 158) and the number of disease types (indolent, significant and aggressive) (|*T*| = 3), that is 1/474 (Kuncheva, 2004).

**Table 2:** Patient numbers within each dataset

Data set	No. patients (%)			
	Indolent	Significant	Aggressive	Total
Clinical data	46 (29.11)	56 (35.44)	56 (35.44)	158
DNA methylation	23 (21.90)	43 (40.95)	39 (37.14)	105
Transcripts 1	20 (21.98)	39 (42.86)	32 (35.16)	91
Transcripts 2	21 (18.26)	48 (41.74)	46 (40.00)	115
Proteomics	34 (29.31)	35 (30.17)	47 (40.52)	116
Glycosylation	41 (35.04)	41 (27.35)	44 (37.61)	117
Full information	10 (21.74)	17 (36.96)	19 (41.30)	46

Table shows the breakdown of sample sizes and indolent, significant and aggressive disease type prevalence across the partially overlapping datasets; the clinical cohort, each of the five datasets and the subset of patients with full clinical and biomarker information.

An advantage of this approach is that it handles the missing data associated with clinical cohorts across multiple biological sources. To make a prediction for new patients, predicted probabilities can be obtained, for the available blocks, using the model coefficients in Table S5 (which are based on the full data in the respective block) and a final classification can be produced by inputting these probabilities into the Geometric Pooling formula above.

#### 2.6.3 Performance assessment

Predictions are made using the Integrated Geometric Pooling (IGP) model and LOOCV predicted probabilities for all 158 patients, even if the absence of information for a given patient from one or more blocks means there are no predictions from the corresponding input biomarker model(s) (Fig. S1). The cross‐tabulation of known disease type against the IGP model's predictions is given in Table S2: 74.68% of patients are classified correctly. However, due to the ordinal multiclass nature of the disease type definition, off‐diagonal corners should be considered more serious misclassifications than other off‐diagonal entries; for instance, labelling an aggressive patient as although they were indolent should be treated as a more serious error than labelling their cancer significant. The classification problem must first be dichotomised into three ‘One vs. All’ binary problems – from indolent vs. not indolent, to aggressive vs. not aggressive at the other extreme – before familiar performance metrics can be calculated.

After dichotomising, three crucial elements of performance assessment can be considered in combination, in each binary scenario: Discriminant Ability (via Receiver Operating Characteristic (ROC) Curve Analysis and AUC scores), Calibration (via Calibration Curve Analysis) and Clinical Benefit (via Decision Curve Analysis). Our previous studies have identified the need to examine Calibration and Clinical Benefit, rather than just ROC Curve Analysis (Boyce *et al*., 2013). These strategies apply to the IGP model just as they do to the individual models.

AUCs can be computed in each of the dichotomised scenarios, and a variety of ways to summarise overall multiclass discriminant ability, such as the Multiple AUC and Ordinal C‐Index (ORC) (Fawcett, 2006; Van Calster *et al*., 2012). The former is a prevalence weighted average of the One vs. All AUCs, while the latter is an unweighted average of the pairwise One vs. One AUCs.

Calibration Curves visualise the degree of concordance between predicted probabilities and actual probabilities. A perfect model is represented by the 45° line (Boyce *et al*., 2013; Steyerberg, 2009). Decision Curves show net benefit according to the model for each disease type as the threshold is varied, *vis‐à‐vis* the strategies of treating either all or no patients of that type as though they were of that type. A model with clinical utility would be above the horizontal Treat‐None line and to the right of the downward‐sloping Treat‐All line (Boyce *et al*., 2013; Vickers and Elkin, 2006).

All work was performed using the statistical software package r, version 3.1.3 (R Core Team, R Foundation for Statistical Computing, Vienna, Austria) using the packages glmnet, plyr, pROC and ROCR (Friedman *et al*., 2010; Robin *et al*., 2011).

### 2.7 Gene ontology enrichment analysis

The list of features in Table S3 was subjected to gene ontology enrichment analysis to identify biological processes with reference to the progression of PCa using PANTHER (PANTHERDB.org/tools/comparetoreflist.jsp). As *P*‐value estimates in Gene Ontology enrichment analysis may be prone to some error, we focused on those associations with *P*‐values less than 0.01 and displaying a great than tenfold enrichment and interpret the findings with some caution but reviewed in the context of the biology identified from other papers.

## 3 Results

### 3.1 Multi‐omics data integration and model resulted in biomarker panel that showed significant improvement over current clinical standard data in distinguishing indolent from aggressive PCa

Using meticulously collected, in accordance with the TRIPOD guideline, biomaterials from patients with well annotated and rigorously documented clinical data (Table S1), we undertook detailed omic analysis to measure the expression of panels of biomarkers. These included DNA methylation, coding and noncoding transcripts, proteins and glycan biomarkers. The resulting extensive and unique dataset comprising patient clinical data and data from the five omic biomarker panels measured in tissue and serum samples from a cohort of 158 patients was analysed by a range of existing and novel statistical approaches.

**Figure 1:**
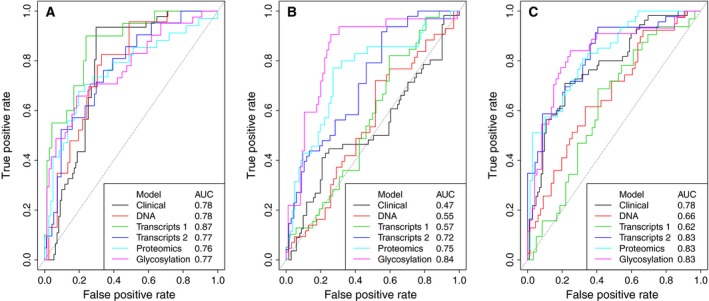
ROC curves for the individual clinical and biomarker panel models and associated AUC values. (A) Indolent vs. nonindolent patients, (B) significant vs. nonsignificant patients, (C) aggressive vs. nonaggressive patients.

The ROC curves with associated One vs. All AUC values for these binary scenarios according to the individual clinical and biomarker panel models are given in Fig. 1 (also see Fig. S2). The individual models performed well with regard to distinguishing indolent from nonindolent and aggressive from nonaggressive patients. Classification difficulty arises when distinguishing between the significant and nonsignificant group; in particular, the AUC for the clinical model of 0.471 indicated low ability to distinguish significant patients.

**Figure 2:**
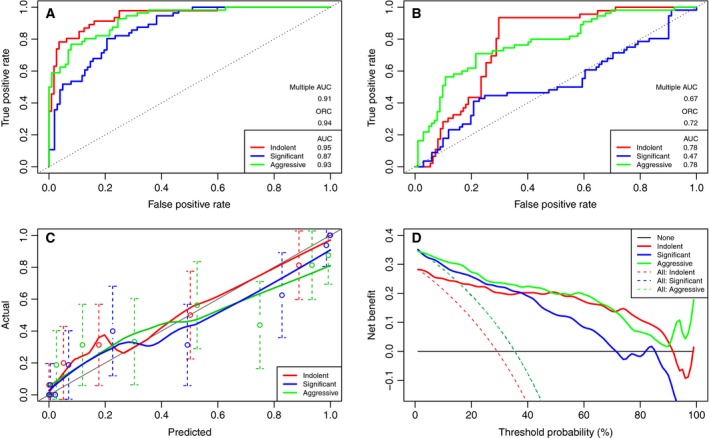
Performance assessment of the IGP model via ROC Curve Analysis (A), calibration curve analysis (C) and decision curve analysis (D). The AUCs in (A) can be compared to those from the ROC curves for the logistic regression model based on the four current clinical parameters of age, Biopsy Gleason, DRE and PSA in (B). Solid colours correspond to indolent, significant and aggressive in all four cases. The horizontal location of the dotted confidence interval error bars in (C) relate to the different bins used in the Calibration Curve Analysis for the indolent, significant and aggressive probabilities.

Although the AUC values from individual panels (Fig. 1) are in line with previous publications, results of the IGP model (Fig. 2) support the hypothesis that multivariate models built from different omic data sources across different biomaterial lead to superior accuracy for prediction over single features.

The IGP model demonstrates excellent discriminant ability, with improvements in the overall discriminant metrics and AUCs for the binary scenarios. The model also demonstrates excellent calibration, across the full range of threshold probabilities for each scenario. Likewise, the model also demonstrates positive net clinical benefit across the range of threshold probabilities (Fig. 2D).

ROC curves from our model compare favourably to those produced by the clinical data alone, in Fig. 1. Further improvements over the current clinical standard – in terms of sensitivity and specificity, and AUCs – are demonstrated in Table 3, wherein the lower limits of the IGP model's 95% confidence intervals for the AUC, Multi AUC and ORC metrics are greater than the upper limits of those belonging to the clinical model. This indicates statistically significant improvements, which also holds true at the 99% confidence level and represents a novel and central finding of this study. Sensitivity and specificity were calculated at the optimal thresholds, achieved at the point of the corresponding ROC curve closest to the theoretical perfect classifier.

**Table 3:** Final Multi AUC and ORC values for the clinical and integrated geometric pooling model

Type	Specificity	Sensitivity	AUCs	Multi AUC	ORC
Clinical Model					
Indolent	0.92 (0.85, 0.97)	0.55 (0.43, 0.66)	0.78 (0.70, 0.85)	0.67 (0.62, 0.72)	0.72 (0.67, 0.78)
Significant	0.67 (0.58, 0.75)	0.45 (0.27, 0.64)	0.47 (0.37, 0.57)		
Aggressive	0.79 (0.70, 0.86)	0.62 (0.48, 0.75)	0.78 (0.70, 0.86)		
Integrated Geometric Pooling Model					
Indolent	0.92 (0.84, 0.96)	0.84 (0.70, 0.93)	0.95 (0.91, 0.98)	0.91 (0.89, 0.94)	0.94 (0.91, 0.96)
Significant	0.82 (0.73, 0.89)	0.67 (0.53, 0.79)	0.87 (0.82, 0.92)		
Aggressive	0.87 (0.79, 0.93)	0.75 (0.62, 0.86)	0.93 (0.89, 0.97)		

The table describes the performance comparison of the clinical model against the IGP model, using various numeric metrics, with 95% Confidence Intervals in parentheses.

**Figure 3:**
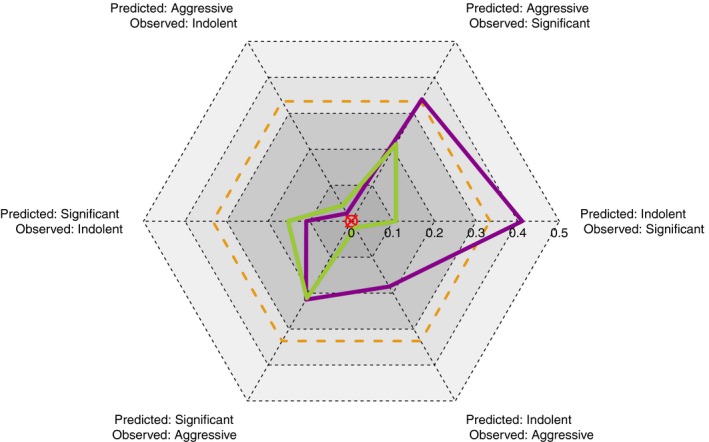
Misclassification Cobweb generated using the misclassification ratios of the confusion ratio matrix, which is the column‐normalised version of the confusion matrix depicting misclassification errors for each of the six (|*T*|^2^ − |*T*|) types of classification error that can be made for this |*T*| = 3‐class problem, depicted by three 6‐sided polygons, which maps the misclassification rates of the confusion ratio matrices resulting from (a) random assignment, whereby a patient is equally likely to be indolent, significant or aggressive, with probability 1/|*T*| = 1/3 (dotted orange); (b) the clinical model (purple); and (c) the IGP model (green).

The improvement over the current clinical standard is clearly visible in the misclassification cobweb (Fig. 3) representing a novel approach to display these data. Given that the best model would be as close as possible to the red centre, the smaller area covered by the green polygon indicates that the IGP model performs not only better than chance performance, but is also a significant improvement over the clinical model. There is a marked improvement in classification accuracy over the clinical model for patients observed to be either significant or aggressive being predicted to be indolent.

Crucially, the IGP model produces a probabilistic classification for all patients using the available blocks and overcomes the problem of missing data which represents another important finding of the paper and a tool for future studies. We have also illustrated that our geometric pooling methodology for model aggregation demonstrates significant performance improvement against analysing biomarkers – or indeed even blocks of biomarkers – in isolation with a Multi AUC value of 0.913 and ORC value of 0.939 (Table 3). This represents a significant improvement on the corresponding value for individual clinical and biomarker block models demonstrated in Fig. 1, thereby proving our initial concept that integrating multiple biomarkers from different biological sources can better risk‐stratify PCa patients. Future research will endeavour to further refine the biomarker panel into a more reduced set of variables that still suffices to distinguish indolent, significant and aggressive disease. Preliminary analyses in this regard are shown in Table S6, detailing the results in terms of Multi AUC and ORC obtained by passing different subsets of blocks, pertaining to clinical, serum‐related and tissue‐related variables, and their combination, through the geometric pooling formula. This table demonstrates that serum alone and serum combined with the clinical variables have the best prediction among the reduced models, with 40 and 44 variables, respectively, which represent the most easily measurable markers to implement as they are based on blood samples, compared to the sampling error associated with biopsy tissue samples.

### 3.2 Gene ontology enrichment analysis showed that biological processes involved in PCa progression are mainly related to stress, metabolic processes and immune response

The table of enriched gene ontology terms is shown in Table S4; the gene ontology terms were filtered to have a minimum of five genes in each entry and a *P*‐value of less than 0.01. The enrichment analysis showed biological processes including response to stress, metabolic processes and immune responses. Table S3 includes the gene/protein name, UniProt identifier, a short description of function for each of the DNA methylation genes, mRNA and peptides found in the panel and a column of their role in PCa and its progression. The suggested associations appear consistent with the literature as discussed in the next section.

## 4 Discussion

Single omic biomarker panels for the identification of indolent and aggressive PCa do exist but have limited accuracy. These include the potential of DNA methylation patterns specifically the DNA hypermethylation MicroArray signature based on 46 genes which indicates poorer survival (Goh *et al*., 2014). Our previous research yielded 17 differentially methylated regions for validation as potential prognostic markers in this study (A. Perry, unpublished data). Blume‐Jensen *et al*. (2015) identified an eight biomarker proteomic assay for intact tissue biopsies predictive of a favourable prostate pathology with an AUC value of 0.68. Studies by Cima *et al*. (2011) have identified a five serum glycoprotein biomarkers signatures for predicting tissue PTEN status and diagnosis and grading that predicted patients with a Gleason score < 7 or > 7 with an AUC value of 0.78. A urine proteomic peptide panel assessed by targeted proteomics identified proteins to predict pT3 disease before radical prostatectomy with an AUC of 0.74 compared to PSA alone of 0.66 (Kim *et al*., 2016). Additional studies by our group have identified a serum protein panel predictive of increased stage of PCa with AUC 0.74 (Fan *et al*., 2011). We further undertook unbiased protein discovery proteomic experiments to identify 59 candidate serum proteins for evaluation which were supplemented with five proteins from the literature (S.R. Pennington, unpublished data) assessed by MRM in the current study. The ability to use miRNA and mRNA targets to distinguish indolent vs. aggressive disease has also received attention (Pickl *et al*., 2014). A 12‐gene tissue expression signature has previously been described in association with aggressive histology in PCa distinguishing low‐grade from high‐grade tumours (Agell *et al*., 2012). For the transcriptomic analysis of the current study, we identified a 33‐gene panel compiled by a comprehensive meta‐analysis of the literature. This list was refined using a bioinformatic computational target validation approach, incorporating data from a pilot global miRNA expression analysis performed by our collaborators in the Harvard School of Public Health. We have also investigated whether differential glycosylation patterns could distinguish between PCa and benign prostate hyperplasia (Saldova *et al*., 2011) using the high‐throughput *N*‐glycan analysis techniques which demonstrated increases in both core‐fucosylated biantennary glycans and α2‐3‐linked sialic acids in the serum *N*‐glycome (Royle *et al*., 2008).

In our current study, we hypothesised that multivariate models built from different omic data (DNA methylation, coding and noncoding transcripts, proteins and glycosylation) across different biomaterials (tissue, serum) would lead to superior accuracy for PCa aggressiveness prediction over singular disease features justified by the fact that PCa is a heterogeneous disease. Our goal was to appropriately risk‐stratify patients and more accurately predict patients with indolent or aggressive disease.

Integrating biomarkers of these different data types as blocks in an aggregated multiblock classifier were shown to add value over analysing them in isolation. This addresses the need for effective predictive integrated multivariate biomarker models in PCa which has been done at the genomic level in breast cancer (Seoane *et al*., 2014). Although the indolent, significant and aggressive disease types are reasonably balanced in terms of the prior prevalences (or relative frequencies) across the full patient cohort, full overlap across the various data blocks could not be achieved, meaning that one or more blocks are missing for some patients and that the prior prevalences within each block are unequal. The geometric pooling methodology used here overcomes the problem of missing information blocks by basing the risk stratification on the full range of available patient information.

Alternative methods for pooling biomarker panels include the Bayesian method proposed by Ankerst *et al*. (2012) were considered because they relax the independence assumption used in the geometric pooling approach. However, this approach makes distributional assumptions within each biomarker panel which did not hold for the panels in our study. Seoane *et al*. (2014) used a number of biomarkers to improve the prediction of breast cancer prognosis using a multiple kernel support vector machine approach which requires all biomarker panels to be available for each subject. The approach taken in the current study facilitated integrating the classification models developed for each individual biomarkers and enabled classification for subjects where biomarker blocks were missing. Thus, predictions were feasible for all subjects in the study.

The classification of patients into indolent, significant and aggressive disease types required the use of classification performance measures that account for the fact that the subjects fall into three classes and that the classes are ordinal in nature. The ORC appropriately accounts for the multiclass and ordinal nature of PCa stratification. The interpretation of ORC in terms of an average of the AUC values for the pairwise classifications yields a measure that can be similarly interpreted as AUC is for binary classification problems.

To evaluate the findings in line with the biology and literature around the individual features, model coefficients (Table S5) were calculated and examined which relate a feature to the probability of a patient exhibiting cancer of a specific indolent, significant or aggressive disease type as follows: A positive coefficient for a given biomarker for a given disease type can be interpreted as a unit change in that same biomarker leading to a change in the same direction of the predicted probability of a patient having cancer of that disease type, whereas negative coefficients cause the predicted probability to move in the opposite direction. A number of important validations of the features were thus identified. For instance, coefficients for the two individual peptides DYVSQFEGSALGK and LLDNWDSVTSTFSK, of −1.33E‐06 and −8.03E‐07, respectively, are consistent with Apolipoprotein A‐1 inverse association with PCa risk (Van Hemelrijck *et al*., 2011). The zinc‐alpha‐2‐glycoprotein, which has a model coefficient value of −1.79E‐01 for aggressive disease, has been shown to be included in a panel of five proteins for the prediction of Gleason 7 or higher (Cima *et al*., 2011). Of the proteins that had two peptides identified the majority had coefficients in agreement between the two peptides; for example, Apolipoprotein E had coefficients of −6.23E‐05 and −9.73E‐06 for significant disease VQAAVGTSAAPVPSDNH and WVQTLSEQVQEELLSSQVTQELR, respectively. However, complement factor H‐related protein 2 had coefficients of 1.17E+00 and −3.71E‐02 with opposing signs for ITCAEEGWSPTPK and TGDIVEFVCK, respectively; this could be due to the presence of multiple proteoforms, post‐translational modifications, variations in proteolytic digestion efficiencies or inaccurate quantification as has previously been described by Kim *et al*. (2016) and would require additional study.

Similar results were found for the Transcripts 1 and 2 panel members – miR.20a.5p has positive coefficient values of 2.58 and 2.20 for significant and aggressive disease, respectively, which is in line with increased tissue expressed with Gleason score 7–10 compared to 6 (Pesta *et al*., 2010; Qiang *et al*., 2014). Likewise, miR101 has positive coefficient values of 0.64 and 3.62 for significant and aggressive disease and a target gene for EZH2 which is a key transcription factor in PCa progression (Lin *et al*., 2016).

Our previous glycosylation study observed significant decreases in triantennary trigalactosylated glycans (including A3G3 and A3G3S3) and tetra‐antennary tetrasialylated outer‐arm‐fucosylated glycans, and increases in tetra‐antennary tetrasialylated structures in men with Gleason score 7 prostate tumours compared with Gleason score 5 cancers (Saldova *et al*., 2011). In the current study, we identified 13 glycosylation peaks two of which were included in the panel – tetra‐antennary tetrasialylated structures and A3G3S3 which also have corresponding coefficient values.

Further evaluation of the panel was carried out using PANTHER gene ontology enrichment analysis which identified differentially expressed features involved in response to stress, metabolic processes and immune responses. These changes in the biological processes of indolent and aggressive disease are reflected in the biomarkers of the selected panel. Previous studies by Lu *et al*. (2016) have identified changes in stress‐related signalling pathways in lethal and nonlethal PCa and suggest that these pathways may be dysregulated in the tumours of men who progress to aggressive and lethal disease. McArdle has also shown the presence of a systemic inflammatory response predicts poor long‐term outcome in localised PCa (McArdle *et al*., 2010) and our own previous work has identified immune response biological pathways in aggressive disease (Fan *et al*., 2011).

## 5 Conclusions

This study is the first to integrate data across clinical data and five omic platforms assessed in tissue and serum which has given a single panel for the classification of indolent and aggressive disease with a significantly improved AUC as compared to clinical features alone or the individual omic AUC values. This clearly supports the hypothesis that the different features across the different platforms and biomaterials adds value to the predictive model. The study identified novel statistical approaches to deal with missing data and integrate these with the assessment of three performance parameters beyond AUC's which can be applied to other diseases. We present evidence to support some members of the panel in the progression of PCa from indolent to aggressive disease and identify new biomarkers for further study. Further cohorts will be required to validate and refine the biomarkers to a panel that can be implemented into clinical practice as the current panel is assessed across a number of omic platforms and is not practical for clinical utility.

## Author contributions

SF, TBM, RJO'K, JO'L, ASP, PMR, SRP, RS and RWW conceived and initiated this study. SB, SF, TBM, RJO'K, JO'L, SRP, ASP, PMR, RS, OS, HS and RWW designed the experiments. LF, SG, CJO'R, CR, ALW and HS carried out the experiments and generated the results. KM and TBM conducted the statistical analysis. SF, LF, SG, TBM, KM, RJO'K, JO'L, CJO'R, SRP, ASP, CR, RS, OS, DCS, ALW and RWW interpreted the results and drew the conclusions. KM, TBM and RWW wrote the initial draft of the manuscript which was reviewed by all co‐authors.

## Supporting information


**Equation S1.** Model equation to estimate the probability of indolent, significant and aggressive disease for each patient.
**Fig. S1.** Heatmaps for indolent, significant and aggressive disease type.
**Fig. S2.** Alternate layout of Fig. 1 information.
**Table S1.** Summary statistics for full Clinical Cohort.
**Table S2.** Cross tabulation of the known indolent, significant and aggressive disease type against the integrated model's predicted classifications.
**Table S3.** Table of gene/protein descriptions in model.
**Table S4.** Gene Ontology table.
**Table S5.** Model coefficients for each feature generated from the logistic regression models.
**Table S6.** Table of different subsets of blocks and resulting Multi AUC and ORC.
