# Supplementary material for: Integrating biomarkers across omic platforms: an approach to improve stratification of patients with indolent and aggressive prostate cancer
Source: Mol Oncol. 2018 Aug 7;12(9):1513–25. doi: 10.1002/1878-0261.12348 (PMC6120220; doi:10.1002/1878-0261.12348)
Supplement: Supplementary file 1 — Equation S1. Model equation to estimate the probability of indolent, significant and aggressive disease for each patient. Fig. S1. Heatmaps for indolent, significant and aggressive disease type. Fig. S2. Alternate layout of Fig. 1 information. Table S1. Summary statistics for full Clinical Cohort. Table S2. Cross tabulation of the known indolent, significant and aggressive disease type against the integrated model's predicted classifications. Table S3. Table of gene/protein descriptions in model. Table S4. Gene Ontology table. Table S5. Model coefficients for each feature generated from the logistic regression models. Table S6. Table of different subsets of blocks and resulting Multi AUC and ORC. [file MOL2-12-1513-s001.pdf]

Supplementary Equation 1:

Model equation to estimate the probability of indolent, significant and aggressive disease for each patient

Equation (*1) above holds when b=B, in the presence of missing data, with unequal prior prevalences, a zero-correction parameter ε, and using the geometric mean of the prevalences from the available blocks. We can look at the simpler cases where b=2 (*2) and b=B (*3) in the presence of full data to see how this is derived

$$
\begin{aligned}
P\left(\mathrm{Type}_{i,t} \mid X_{i,Agg}, M_{Agg}\right) &= P\left(\mathrm{Type}_{i,t} \mid X_{i,Agg}, M_1, M_2\right) \\
&\propto P\left(X_{i,Agg}, M_1, M_2 \mid \mathrm{Type}_{i,t}\right) P\left(\mathrm{Type}_{i,t}\right) \\
&= P\left(X_{i,1} M_1 \mid \mathrm{Type}_{i,t}\right) P\left(X_{i,2} M_2 \mid \mathrm{Type}_{i,t}\right) P\left(\mathrm{Type}_{i,t}\right) \\
&\propto \frac{P\left(\mathrm{Type}_{i,t} \mid X_{i,1}, M_1\right) P\left(\mathrm{Type}_{i,t} \mid X_{i,2}, M_2\right)}{P\left(\mathrm{Type}_{i,t}\right)} \qquad \text{(when p = 2)} \\
&\propto \frac{\prod_{p=1}^{P} P\left(\mathrm{Type}_{i,t} \mid X_{i,p}, M_p\right)}{P\left(\mathrm{Type}_{i,t}\right)^{p-1}} \qquad \text{(when p = P)}
\end{aligned}
$$

Supplementary Figure 1: Heatmaps for indolent, significant and aggressive disease type

Heatmaps for indolent (A), significant (B) and aggressive (C) disease type showing the probabilities of correct classification according to the clinical, various biomarker, and integrated models. Grey regions correspond to patients with missing information, otherwise the darker the region the greater the probability. Crucially, the IGP model produces a probabilities classification for all patients using the available blocks, and overcomes the problem of missing data.

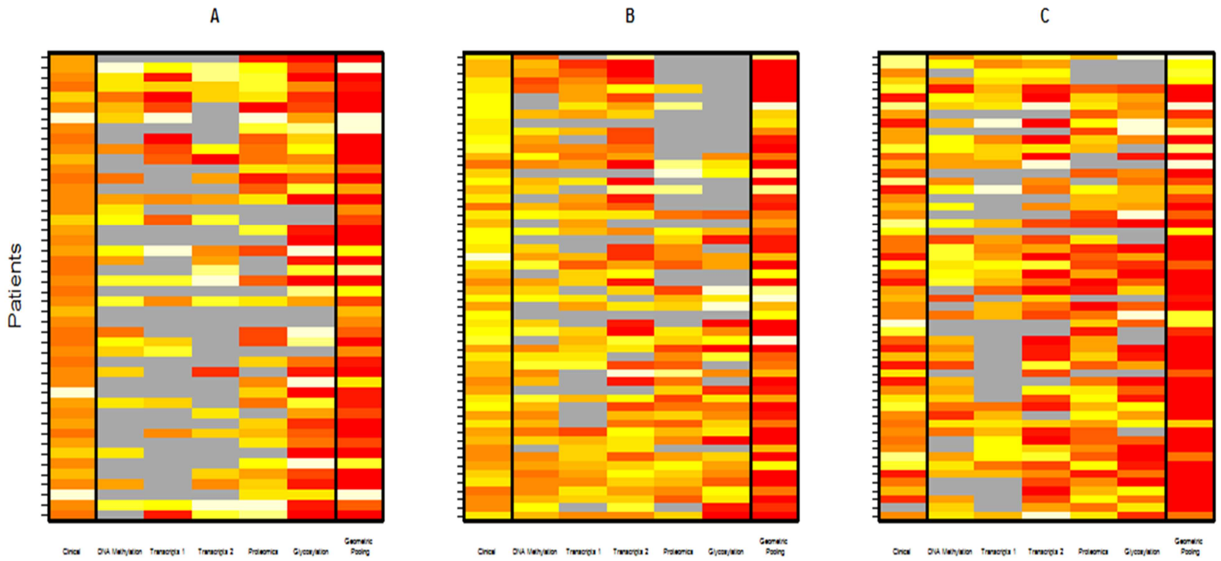

Supplementary Figure 2: Alternate layout of Figure 1 information.

ROCs for the individual clinical and biomarker panel models, with associated AUC values.

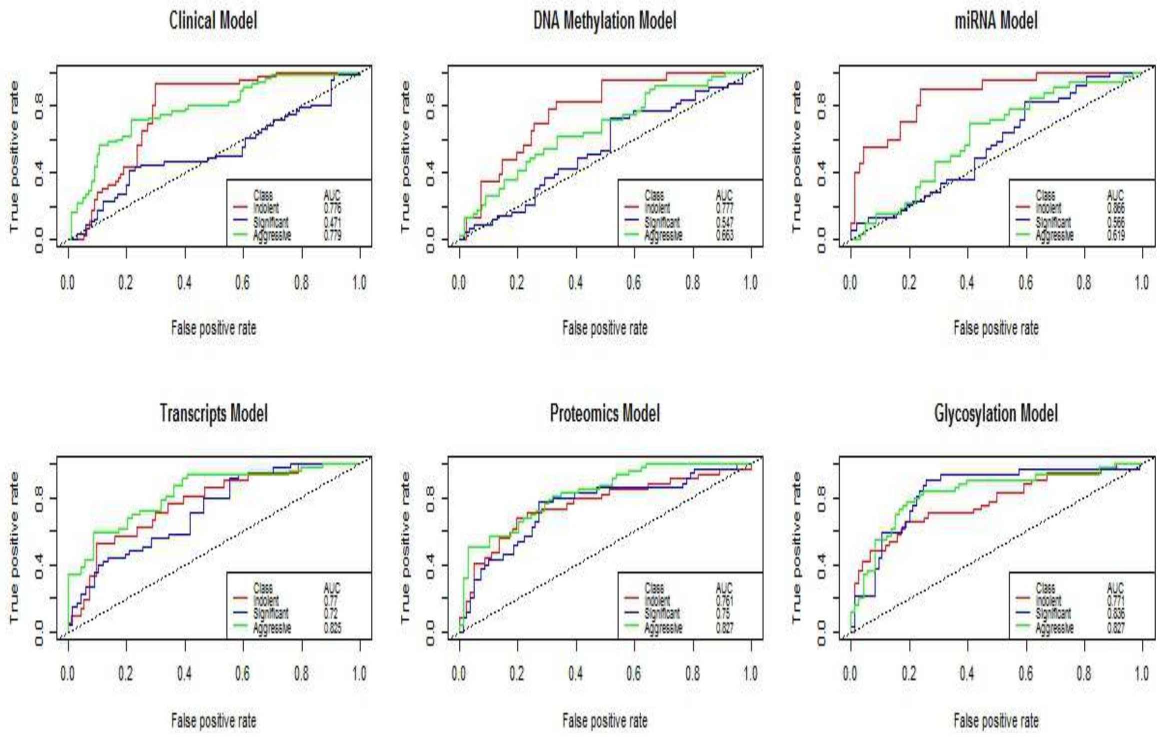

Supplementary Table 1: Summary statistics for full Clinical Cohort

Pre-operative radical prostatectomy PSA – Pre-operative PSA, Digital rectal examination – DRE, Year – Y, Gleason score – GS.

	**Full Cohort** (n = 158)	**Indolent** (n = 46)	**Significant** (n = 56)	**Aggressive** (n = 56)	**p**
**Age (y)**					
Mean (Median)	60.6 (61)	59.8 (60)	59.8 (60)	62 (62.5)	0.087
Range	45 - 74	49 - 70	48 - 73	45 - 74	
**Pre-Op PSA (ng/ml)**					
Mean (Median)	6.89 (6.45)	6.38 (6)	6.72 (6.35)	7.47 (6.85)	0.168
Range	0.7 - 18.7	0.7 - 12.3	2.4 - 14.7	2.3 - 18.7	
**Clinical Stage-DRE (%)**					
T1c	79 (50)	26 (57)	31 (55)	22 (39)	0.082
T2a	36 (23)	5 (11)	14 (25)	17 (30)	
Not Described	43 (27)	15 (33)	11 (20)	17 (30)	
**Biopsy GS (%)**					
≤ 6	76 (48)	40 (93)	23 (41)	10 (18)	0.000
3 + 4 = 7	52 (33)	3 (7)	25 (45)	24 (43)	
4 + 3 = 7	18 (11)	0 (0)	7 (13)	11 (20)	
≥ 8	11 (7)	0 (0)	1 (2)	10 (18)	
Not Described	1 (1)	0 (0)	0 (0)	1 (2)	
**Prostatectomy Stage**					
OC	102 (65)	46 (100	56 (100)	0 (0)	0.000
NOC	56 (35)	0 (0)	0 (0)	56 (100)	
**Prostatectomy GS (%)**					
≤ 6	47 (30)	46 (100	1 (2)	0 (0)	0.000
3 + 4 = 7	72 (46)	0 (0)	46 (82)	26 (46)	
4 + 3 = 7	26 (16)	0 (0)	5 (9)	21 (38)	
≥ 8	13 (8)	0 (0)	4 (7)	9 (16)	

Supplementary Table 2: Cross tabulation of the known indolent, significant and aggressive disease type against the integrated model's predicted classifications

		Predicted		
		Indolent	Significant	Aggressive
	Indolent	37	7	2
	Significant	6	38	12
Observed	Aggressive	1	12	43

Supplementary Table 3: Table of gene/protein descriptions in model

Table of gene/protein names, UniProt identifier, short description of each peptide, DNA methylated gene and mRNA identified in the panel. Data gathered from UniProt (www.uniprot.org)

Supplementary Table S5: Table of gene/protein names, UniProt identifier, short description of each peptide, DNA methylated gene and mRNA identified in the panel. Data gathered from UniProt (www.uniprot.org)						
Proteomics	**Peptide Sequence/gene name**	**Protein Name from UniProt**	**Gene Names from UniProt**	**Unique Identifier from UniProt**	**Uniprot Function and Biological Role**	**Role related to PCa**
	DYVSQFEGSALGK LLDNWDSVTSTFSK	Apolipoprotein A-I	APOA1	P02647	Participates in the reverse transport of cholesterol from tissues to the liver for excretion by promoting cholesterol efflux from tissues and by acting as a cofactor for the lecithin cholesterol acyltransferase (LCAT). As part of the SPAP complex, activates spermatozoa motility. Lipid metabolism, promotes fat efflux including cholesterol	APOA1 is inversely associated with Pca risk

	EPCVESLVSQYFQTVTDYGK	Apolipoprotein A-II	APOA2	P02652	May stabilize HDL (high density lipoprotein) structure by its association with lipids, and affect the HDL metabolism. Alters the intermediate HDL metabolism in opposing ways by increasing LCAT or decreasing HL the atherogenicity of lipid metabolism	APOPA2 is overexpressed in prostate tumours and serum from individuals with Pca as well as patients with normal PSA.
	IDQNVEELK	Apolipoprotein A-IV	APOA4	P06727	May have a role in chylomicrons and VLDL secretion and catabolism. Required for efficient activation of lipoprotein lipase by ApoC-II; potent activator of LCAT. Apoa-IV is a major component of HDL and chylomicrons. Display anti-oxidant and anti-atherogenic propertics in rodent	

					models.	
	NPNLPPETVDSLK	Apolipoprotein D	APOD	P05090	APOD occurs in the macromolecular complex with lecithin-cholesterol acyltransferase. It is probably involved in the transport and binding of bilin. Appears to be able to transport a variety of ligands in a number of different contexts. Component of high-density lipoprotein, link between HDL and LDL	APOD has been shown to be expressed in high grade PIN and Pca but not in non-malignant epithelial cells so considered a marker of malignant transformation.

	WVQTLSEQVQEELLSSQVTQELR VQAAVGTSAAPVPSDNH	Apolipoprotein E	APOE	P02649	Mediates the binding, internalization, and catabolism of lipoprotein particles. It can serve as a ligand for the LDL (apo B/E) receptor and for the specific apo-E receptor (chylomicron remnant) of hepatic tissues. Is produced by macrophages - secretion is down regulated by inflammatory cytokines and upregulated by TGF-beta. Potential inhibitor of cell proliferation, immune regulation and modulation of differentiation.	APOE expression correlates directly with Gleason Score in tissue section, hormone independent as well as local and distant invasive PCa - May represent a marker of more aggressive cells in PCa

	DLLLPQPDLR VAAGAFQGLR	Leucine rich a-2-glycoprotein	LRG1	P02750	The function of LRG1 remains largely unknown, although reports have predicted its role in cell adhesion due to its leucine-rich repeats, granulocytic differentiation due to its expression in neutrophil lineage experiments, and cell migration due to its overexpression in high-endothelial venules and tendency to bind extracellular matrix proteins. LRG1 has been implicated as a protein involved upstream of the TGF-βR II pathway, suggesting a role in signalling. Serum LRG1 binding to cytochrome c has been recently demonstrated and is proposed to play a role in cell survival and apoptosis. It has	LRG1 has been shown to be a blood-based biomarker for aggressive prostate cancer able to enhance prostate cancer risk stratification.

					been shown to be involved in EMT.	
	ITCAEEGWSPTPK TGDNEFVCK	complement factor H-related protein 2	CFHR2	P36980	Involved in complement regulation. The dimerized forms have avidity for tissue-bound complement fragments and efficiently compete with the physiological complement inhibitor CFH. Can associate with lipoproteins and may play a role in	

					lipid metabolism	
	SDLAVPSELALLK<br>AAIPSALDTNSSK	Galectin-3-binding protein	LGALS3BP	Q08380	Promotes intergrin-mediated cell adhesion. May stimulate host defense against viruses and tumour cells. LGALS3BP, has been implicated in a variety of biological functions including cell proliferation, apoptosis, angiogenesis, tumour progression, and metastasis. Candidates for the binding proteins include PSA and ZAG. LGALS3BP was able to inhibit neutrophil activation that might be important for immune evasion of	LGALS3BP has been observed to be up-regulated in human colorectal and PCa specimens.

					tumour cells during cancer progression.	
	SVLGQLGITK	Alpha-1-antitrypsin	SERPINA1	P01009	Inhibitor of serine proteases. Its primary target is elastase, but it also has a moderate affinity for plasmin and thrombin. Irreversibly inhibits trypsin, chymotrypsin and plasminogen activator. The aberrant form inhibits insulin-induced NO synthesis in platelets, decreases coagulation time and	

					has proteolytic activity against insulin and plasmin. A marker of inflammation - has a broad anti-inflammatory spectrum.	
	ADLSGITGAR	Alpha-1-antichymotrypsin	SERPINA3	P01011	Although its physiological function is unclear, it can inhibit neutrophil cathepsin G and mast cell chymase, both of which can convert angiotensin-1 to the active angiotensin-2. an acute phase protein, forms a complex with PSA	Part of a tissue gene signature that could predict lethal PCa (2).

	NEDSLVFVQTDK	Alpha-2-macroglobulin	A2M	P01023	Is able to inhibit all four classes of proteinases by a unique 'trapping' mechanism. Functions as a universal protease inhibitor in serum and is capable of binding various cytokines and growth factors. - complexs to PSA.	α2M in elevated concentrations results in decreased PHA-induced lymphocyte response and suggest that α2M may have immunoregulatory properties in advanced PCa
	DFDFVPPVVR	Complement C3	C3	P01024	C3 plays a central role in the activation of the complement system. Its processing by C3 convertase is the central reaction in both classical and alternative complement pathways. After activation C3b can bind covalently, via its reactive thioester, to cell surface carbohydrates or immune aggregates	

	TEHYEEQIEAFK	Complement component C9	C9	P02748	Constituent of the membrane attack complex (MAC) that plays a key role in the innate and adaptive immune response by forming pores in the plasma membrane of target cells. C9 is the pore-forming subunit of the MAC	
	ELGCGAASGTPSGILYEPPAEK	CD5 antigen-like	CD5L	O43866	Secreted protein that acts as a key regulator of lipid synthesis: mainly expressed by macrophages in lymphoid and inflammed tissues and regulates mechanisms in inflammatory responses, such as infection or atherosclerosis. Also acts as an inhibitor of apoptosis in macrophages: promotes macrophage survival	

					from the apoptotic effects of oxidized lipids in case of atherosclerosis.	
	EDSLEAGLPLQVR	Chromogranin-A	CHGA	P10645	Chromogranin A (CgA) is an acidic glycoprotein that is commonly expressed by neuroendocrine cells and constitutes one of the most profuse components of secretory granules. When a tumour develops in an endocrine tissue, it becomes the main source of circulating CgA. Its concentration is thought to be elevated in relation to neuroendocrine	

					differentiation of prostate cancer.	
	TTLSGAPCQPWASEATYR	Coagulation factor XII	F12	P00748	Factor XII is a serum glycoprotein that participates in the initiation of blood coagulation, fibrinolysis, and the generation of bradykinin and angiotensin. Prekallikrein is cleaved by factor XII to form kallikrein, which then cleaves factor XII first to alpha-factor XIIa and then trypsin cleaves it to beta-factor XIIa. Alpha-factor XIIa activates factor XI to factor XIa	

	YGIDWASGR	Ficolin-3	FCN3	O75636	May function in innate immunity through activation of the lectin complement pathway. Calcium-dependent and GlcNAc-binding lectin. Has affinity with GalNAc, GlcNAc, D-fucose, as mono/oligosaccharide and lipopolysaccharides from S.typhimurium and S.minnesota.	
	LAAIAESGVER	Proteasome subunit beta type 6	PSMB6	P28072	The proteasome is a multicatalytic proteinase complex which is characterized by its ability to cleave peptides with Arg, Phe, Tyr, Leu, and Glu adjacent to the leaving group at neutral or slightly basic pH. The proteasome has an ATP-dependent	No expression in PCa epithelial cells but was expressed in the surrounding stroma and inflammatory cells. Also expression at higher levels in the serum of PCa patients.

					proteolytic activity. This unit is responsible of the peptidyl glutamyl-like activity. May catalyze basal processing of intracellular antigens	
	ETLLQDFR	Protein AMBP	AMBP	P02760	Inter-alpha-trypsin inhibitor inhibits trypsin, plasmin, and lysosomal granulocytic elastase. Inhibits calcium oxalate crystallization	Concentrations of AMPB in urine was significantly higher in PCa.
	WEAEPVYVQRP	Zinc alpha-2-glycoprotein	AZGP1	P25311	Stimulates lipid degradation in adipocytes and causes the extensive fat losses associated with some advanced cancers. May bind polyunsaturated fatty acids	AZGP1 expression in tissue is decreased in patients who progress to biochemical recurrance.

	NVPLPVIAELPPK	Ig mu chain C region	IGHM	P01871	IgM antibodies play an important role in primary defense mechanisms. They have been shown to be involved in early recognition of external invaders like bacteria and viruses, cellular waste and modified self, as well as in recognition and elimination of precancerous and cancerous lesions. It may cause death of cells by apoptosis.	
	EAVPEPVLLSR	Transforming growth factor beta 1	TGFB1	P01137	Multifunctional protein that controls proliferation, differentiation and other functions in many cell types. Many cells synthesize TGFB1 and have specific receptors for it. It positively and negatively regulates many other growth factors. Can induce	Loss of TGFB1 promotes PCa metastasis.

					epithelial-to-mesenchymal transition (EMT) and cell migration in various cell types.	
DNA Methylation	GSTP1	glutathione S-transferase	GSTP1	P09211	Phase II drug detoxifying enzymes that play an essential role in the maintenance of cell integrity and protection against DNA damage by catalyzing the conjugation of glutathione to a wide variety of exo- and endogenous electrophilic substrates.	GSTP1 methylation is associated with the development and progression of PCa

	CTNNA2	catenin alpha 2	CTNNA2	P26232	May function as a linker between cadherin adhesion receptors and the cytoskeleton to regulate cell-cell adhesion and differentiation in the nervous system. Regulates morphological plasticity of synapses and cerebellar and hippocampal lamination during development. Functions in the control of startle modulation	
	MAGPIE.1	non-coding DNA				

	LXN	Latexin	LXN	Q9BS40	Mouse haematopoietic stem cell (SC) regulator. Stem cell control gene. Strong effect on the fate of the tumour initiating cells in prostate cancer. For example, when latexin is expressed in a tumour initiating cell, the cell loses its tumour initiating properties, and its ability to migrate and invade.	LXN expression was inversely correlated with taxane resistance in a panel of PCa cell lines. LXN knockdown conferred docetaxel resistance to PCa cells in *vitro and in vivo*, whereas LXN overexpression reduced docetaxel resistance in several PCa cell lines. LXN is silenced in the stem cell fraction of primary prostate epithelial cells, and that expression increases as cells differentiate through the prostate basal epithelial hierarchy towards mature luminal

						cells.
Transcripts (mRNA)	ALCAM	Activated leukocyte cell adhesion molecule / CD166 antigen	ALCAM	Q13740	CD166 antigen is a 100-105 kD typeI transmembrane glycoprotein that is a member of the immunoglobulin superfamily of proteins. CD166 plays an important role in mediating adhesion interactions between thymic epithelial cells and CD6+ cells during	ALCAMs presence in the blood corresponded to the presence of malignant, metastatic prostate tumours in mice. ALCAM up-regulation was found in most low-grade tumours (Gleason grade 1–3),

					intrathymic T cell development. Recently, CD166 has also been used as a potential cancer stem cell marker. TGFb responsive marker	whereas down-regulation occurred preferentially in high-grade tumours (Gleason grade 4 and 5)
	FAM49B	Family with sequence similarity 49 member B	FAM49B	Q9NUQ9		
	IGFBP3	Insulin Growth factor binding protein 3	IGFBP3	P17936	IGF-binding proteins prolong the half-life of the IGFs and have been shown to either inhibit or stimulate the growth promoting effects of the IGFs on cell culture. They alter the interaction of IGFs with their cell surface receptors. Also exhibits IGF-independent	IGF axis plays and important role in PCa development and progression

					antiproliferative and apoptotic effects mediated by its receptor TMEM219/IGFBP-3R	
	AMACR	Alpha-methylacyl-CoA racemase	AMACR	Q9UHK6	Racemization of 2-methyl-branched fatty acid CoA esters. Responsible for the conversion of pristanoyl-CoA and C27-bile acyl-CoAs to their (S)-stereoisomers. This protein is involved in the pathway fatty acid metabolism, which is part of Lipid metabolism.	AMACR contributed to PCa risk and tissue overexpression was found in PCa.

	SFRP4	Secreted frizzled-related protein 4	SFRP4	Q6FHJ7	Soluble frizzled-related proteins (sFRPS) function as modulators of Wnt signaling through direct interaction with Wnts. They have a role in regulating cell growth and differentiation in specific cell types. SFRP4 may act as a regulator of adult uterine morphology and function. Increases apoptosis during ovulation possibly through modulation of FZ1/FZ4/WNT4 signaling (By similarity). Has phosphaturic effects by specifically inhibiting sodium-dependent phosphate uptake	Higher SFRP4 expression in PCa compared with normal samples, and in high (3–5) compared with low (1–2) Gleason grade. SFRP4 expression was a significant predictor of biochemical recurrence.

Supplementary Table 4: Gene Ontology table

Generated from PANTHERDB.org/tools/comparetoreflist.jsp.

GO biological process complete	Number	Expected	Fold Enrichment	P value
negative regulation of biological process	21	6.54	3.21	0.000187
negative regulation of cellular process	19	6.05	3.14	0.00273
regulation of response to stimulus	19	5.31	3.58	0.000311
regulation of biological quality	17	5.07	3.36	0.00775
response to stress	20	4.81	4.16	0.00000606
regulation of protein metabolic process	15	3.62	4.14	0.00357
negative regulation of metabolic process	15	3.56	4.21	0.0029
negative regulation of cellular metabolic process	14	3.3	4.24	0.0082
negative regulation of macromolecule metabolic process	14	3.28	4.27	0.00746
single-organism transport	14	3.05	4.59	0.0031
negative regulation of response to stimulus	13	2	6.49	0.000176
vesicle-mediated transport	12	1.84	6.51	0.000725
defense response	14	1.8	7.77	0.00000379
negative regulation of molecular function	12	1.58	7.58	0.000137
negative regulation of protein metabolic process	11	1.49	7.39	0.000838
negative regulation of cellular protein metabolic process	11	1.4	7.83	0.000468
regulation of immune response	10	1.28	7.84	0.0022
negative regulation of catalytic activity	11	1.21	9.06	0.000106
regulation of response to external stimulus	10	1.17	8.51	0.00102
regulation of defense response	10	1.11	9.03	0.000592
regulation of proteolysis	10	1.02	9.8	0.000275
inflammatory response	8	0.69	11.58	0.00244
negative regulation of hydrolase activity	8	0.58	13.74	0.000667
regulation of inflammatory response	8	0.45	17.64	0.0000983
exocytosis	7	0.45	15.49	0.0022
regulation of lipid metabolic process	8	0.42	18.96	0.0000565
negative regulation of endopeptidase activity	6	0.34	17.4	0.00895
regulated exocytosis	7	0.32	21.56	0.000236
regeneration	7	0.26	27.19	0.0000487
negative regulation of immune response	5	0.18	27.96	0.00785
protein activation cascade	6	0.16	38.48	0.0000873
platelet degranulation	6	0.15	39.57	0.000074
regulation of acute inflammatory response	5	0.11	45.99	0.000685

Supplementary Table 5: Model coefficients for each feature generated from the logistic regression models

Model Coefficients			
Variable Name	Indolent	Significant	Aggressive
Clinical Model (Missing DRE Baseline)			
Intercept	-	-1.998083	-6.123888
Age	-	0.01453979	0.0703767
bxGS (3+4)	-	2.706664	3.704523
bxGS (4+3)	-	12.99801	14.27869
bxGS (8)	-	11.88034	14.95097
DRE (-ve)	-	0.241635	-0.6509732
DRE (+ve)	-	0.59605324	0.08039543
PSA	-	0.04404586	0.09580916
DNA Methylation Model			
Intercept	-	-1.263485	-1.754118
CTNNA2.	-	-0.6639964	0.3091123
GSTP1	-	0.3228433	0.2445532
LXN	-	0.2711671	0.4025234
MAGPIE.1	-	24.44606	24.50886
miRNA Model			
Intercept	-	-1.218955	-5.065251
miR.143.3p	-	1.033509	1.832915
miR.20a.5p	-	2.582877	2.203067
miR.221.3p	-	-1.080718	-1.801422
miR.663a	-	-0.6205074	-0.8686141
Transcripts Model			
Intercept	-	1.192922	1.43363
ALCAM	-	2.2223242	-0.5366789
AMACR	-	1.925648	1.030865
FAM49B	-	-2.505785	1.745143
IGFBP3	-	-1.590281	-2.744171
SFRP4	-	-0.7919851	2.6942815
miR.101	-	0.6419335	3.6224854
miR.16.1	-	-0.3889219	-1.8015431
miR.222	-	-0.4104966	-2.0850237
mir.330	-	-0.1986042	-1.4290306

Proteomics Model			
Intercept	-1.36E+00	9.80E-01	3.81E-01
AAIPSALDTNSSK Present	1.49E-01	-	-
ADLSGITGAR	8.43E-05	-	-
DFDFVPPVVR	-	-	2.15E-06
DLLLPQPDLR	-	-2.01E-05	-
DYVSQFEGSALGK	-	-1.33E-06	-
EAVPEPVLLSR Present	-	-	1.24E+00
EDSLEAGLPLQVR Present	-6.17E-02	-	-
ELGCGAASGTPSGILYEPPAEK Present	-	-	-1.06E+00
EPCVESLVSQYFQTVTDYGK	-	-	5.25E-06
ETLLQDFR	-	-	1.01E-05
IDQNVEELK	-	-	4.64E-05
ITCAEEGWSPTPK Present	1.17E+00	-	-1.04E-01
LAAIAESGVER Present	-6.91E-02	-	-
LLDNWDSVTSTFSK	-	-8.03E-07	1.90E-06
NEDSLVFVQTDK	-3.46E-05	-	-
NPNLPPETVDSLK	3.76E-06	-	-
NVPLPVIAELPPK	-	6.08E-06	-
SDLAVPSELALLK	-	-	-5.16E-06
SVLGQLGITK	-	-	9.93E-08
TEHYEEQIEAFK	-	5.05E-04	-
TGDIVEFVCK Present	-3.71E-02	-	-
TTLSGAPCQPWASEATYR Present	-	-	-2.68E-01
VAAGAFQGLR	-	-	1.11E-05
VQAAVGTSAAPVPSDNH	-	-6.23E-05	-
WEAEPVYVQR Present	-	-	-1.79E-01
WVQTLSEQVQEELLSSQVTQELR	-	-9.73E-06	-
YGIDWASGR	2.50E-04	-	-
Glycosylation Model			
Intercept	-	14.352661	-7.095152
A1	-	-17.29343	-18.85115
A2[6]BG1	-	14.6619	46.84638
FA2[6]BG1	-	-10.098118	-3.198295
M6 D3	-	2.754333	-8.022708
A2[3]G1S[3]1	-	45.748588	-9.037065
FA2BG2	-	10.82362	9.115197
A2BG2S[6]1	-	-15.150946	8.025789
A2G2S[3]1	-	-2.867494	1.728924

A2BG2S[3,6]1	-	2.815797	-4.131
FA2G2S[3,6]2	-	-0.57749761	0.03206105
A3G3S[3,3,3]3	-	-6.07707	-3.385527
A4F1G3S3	-	2.661669	-6.359662
A4G4S[3,3,3,3]4	-	1.505273	16.760512

Supplementary Table 6: Table of different subsets of blocks and resulting Multi AUC and ORC

Results in terms of Multi AUC and ORC obtained by passing different subsets of blocks, pertaining to clinical, serum-related and tissue-related variables, and their combination, through the geometric pooling formula. The number of markers retained in each subconfiguration is also given.

Blocks	# Markers	Multi AUC	ORC
All	61	0.913	0.939
Clinical Only	4	0.669	0.724
Tissue Only	17	0.801	0.843
Serum Only	40	0.845	0.872
Clinical + Tissue	21	0.832	0.872
Clinical + Serum	44	0.874	0.910
Tissue + Serum	57	0.893	0.922
